# Helminth Fauna in Captive European Gray Wolves (*Canis lupus lupus*) in Germany

**DOI:** 10.3389/fvets.2017.00228

**Published:** 2017-12-22

**Authors:** Johanna Daniela Bindke, Andrea Springer, Michael Böer, Christina Strube

**Affiliations:** ^1^Institute for Parasitology, University of Veterinary Medicine Hannover, Hannover, Germany; ^2^Zoo Osnabrueck, Osnabrueck, Germany

**Keywords:** helminths, coproscopy, zoo, zoological garden, zoonosis, Taeniidae, *Toxocara* spp., hookworms

## Abstract

Captive as well as free-ranging wolves, which are currently recolonizing Germany, may harbor a variety of gastrointestinal parasites. This study investigated endoparasites in captive European gray wolves (*Canis lupus lupus*) using coproscopical methods. Fecal samples were collected monthly between October 2012 and November 2013 from 18 wolf enclosures in 14 German zoological gardens, representing 72 individual wolves. In total, 1,041 fecal samples including 26 bulk samples were analyzed by the sedimentation and flotation method. The most frequently detected egg morphotypes included five nematodes [Ancylostomatidae (*Ancylostoma* or *Uncinaria* spp.), *Toxocara canis, Toxascaris leonina, Trichuris vulpis*, and *Capillaria*/*Eucoleus* spp.], one cestode (Taeniidae) and one trematode (*Alaria alata*). 44.76% of all samples were positive for at least one of these egg morphotypes. Overall, Ancylostomatidae showed the highest frequency (30.84% of all samples), followed by *Capillaria/Eucoleus* spp. (19.88%), *Toxocara canis* (5.19%), taeniids (3.75%), *Trichuris vulpis* and *Alaria alata* (3.65% each), and *Toxascaris leonina* (1.25%). As fecal samples were collected from the environment and could not be assigned to individual wolves, sample results were combined per zoo and month. General linear mixed models were employed to analyze the effect of season and management factors on the occurrence of Ancylostomatidae, *Capillaria/Eucoleus* spp., *Toxocara canis* and taeniids. No statistically significant effect of season was found, whereas anthelmintic treatment negatively affected Ancylostomatidae egg excretion. Detected parasites and their prevalences are comparable to previous studies on wolf parasitism conducted elsewhere in Europe. As many of the most prevalent helminths are of zoonotic importance, routine anthelmintic treatment of captive wolves should be recommended.

## Introduction

Since 2000, the European gray wolf (*Canis lupus lupus*) has begun to recolonize Germany. The first packs returned from Poland and have established sustainable populations in the federal states of Brandenburg, Mecklenburg–Western Pomerania, Lower Saxony, Saxony, and Saxony-Anhalt (1). In addition, wolves are kept in many zoological gardens, with European gray wolves (*Canis lupus lupus*), eastern timber wolves (*Canis lupus lyacon*), and tundra wolves (*Canis lupus albus*) as the most commonly kept subspecies (2).

Wolves may harbor a variety of parasites with relevance for domestic animals and even human health. For example, both wild and domestic carnivores may act as definitive hosts for cestodes of the family Taeniidae, which may be responsible for morbidity and mortality in their intermediate hosts. *Taenia ovis, Taenia hydatigena*, and *Taenia multiceps*, for example, are causative agents of cysticercosis in domestic ruminants, which may compromise herd health and lead to production losses as affected organs or carcasses need to be condemned (3). *Echinoccocus granulosus* and *Echinococcus multilocularis*, the causative agents of cystic and alveolar echinococcosis, respectively, are of considerable zoonotic importance, causing potentially fatal disease in humans (4). Furthermore, juvenile stages of the trematode *Alaria alata* reside in muscle and fatty tissue of paratenic hosts, including humans (5). Zoonotic nematodes of canines include, amongst others, the hookworm *Ancylostoma caninum*, which may cause the so-called *larva migrans cutanea* in humans, and the roundworm *Toxocara canis*, whose infective larvae are responsible for several forms of human toxocarosis, namely *larva migrans visceralis*, ocular toxocarosis, neurotoxocarosis, and covert toxocarosis (6). Furthermore, wolves may be hosts to zoonotic protozoa, e.g., *Giardia duodenalis* (7, 8), which may cause enteritis in humans.

Since humans are in quite close contact with animals and their feces in zoos or wildlife parks, captive wolves may pose a potential health risk to animal caretakers. However, also the captive wolves themselves may suffer from parasite infections. Thus, the aim of this study was to describe the helminth fauna of captive European gray wolves (*Canis lupus lupus*) in Germany. So far, only a few studies exist on the endoparasite fauna of captive wolves in Central Europe. In one study, endoparasites of North American large carnivores, including wolves, in German zoological gardens were described based on zoo health records (9). Another study compared the endoparasite fauna of wild and captive wolves in Poland (10). However, to the best of our knowledge, no study has investigated year-round endoparasite burdens of captive European gray wolves in Germany to assess seasonal patterns. Seasonal differences in helminth egg excretion have been described in several canids, including wild foxes (11) and domestic dogs (12). In wild wolves, seasonal parasite patterns have been mainly attributed to variations in resource use or the presence of susceptible pups (7). In addition, changes in host immunity or the endocrinological status, e.g., related to reproduction, might affect parasite excretion patterns (12); however, evidence regarding this phenomenon in wolves is lacking. To investigate whether or not seasonal patterns in helminth egg excretion occur in captive wolves, a generalized linear-mixed model (GLMM) analysis was included in this study. Furthermore, questionnaire-based data were analyzed to assess possible management factors influencing egg excretion.

## Materials and Methods

### Study Design and Fecal Sample Collection

Fourteen public and private zoos and wildlife parks (hereafter referred to as zoos) keeping European gray wolves (*Canis lupus lupus*) distributed across Germany participated in this study. Fecal samples were collected from the ground of the enclosures in monthly intervals from October 2012 to November 2013. Only fresh fecal samples were collected as determined by the presence of a mucus layer and a characteristic smell. As wolves lived in groups and could not be separated, fecal samples could not be assigned to single individuals. Collected samples were transported to the Institute of Parasitology of the University of Veterinary Medicine Hannover for coproscopical examinations.

To gain information on the enclosure size, number, age, and sex of the wolves living in the fenced area, type of husbandry (single or mixed-species exhibit), potential offspring in 2012 or 2013, origin and composition of the wolves’ food, antiparasitic treatment regime and whether dogs were allowed in the park or not, a questionnaire was provided to the zoos.

### Coproscopical Examinations

Fecal samples sent by the zoos were weighed and preserved at −20°C until coproscopical examination. First, 5 g of each sample was screened for the presence of parasite eggs by use of the combined sedimentation-flotation method using zinc sulfate solution [ZnSO4, specific gravity (SG) 1.30] as flotation solution and centrifugation at 450 × *g* to enhance flotation as described by Becker et al. (13). If parasite eggs were detected, the McMaster method was carried out to determine the number of eggs per gram feces (EPG). An amount of 4 g feces was processed with 60 ml flotation solution [saturated NaCl solution (SG 1.2) for hookworm and roundworm eggs; zinc sulfate solution (SG 1.3) for trichurid, capillariid, taeniid, and *A. alata* eggs] as described by Becker et al. (13) except that four McMaster counting grids were examined. Thus, a sensitivity of 25 EPG was achieved. Presence of coccidian oocysts was noted but not included in subsequent analyses since unsporulated oocysts may not necessarily originate from the wolves, but may represent intestinal passengers originating from food animals, and, furthermore, differentiation of sporulated oocysts was not sufficiently possible due to degradations following the freeze-thaw process.

### Statistical Analyses

Generalized linear-mixed models with binomial error structure and logit link function were used to analyze which factors influenced the probability of presence/absence of the most prevalent parasite species (Ancylostomatidae, *Capillaria/Eucoleus* spp., Taeniidae, and *Toxocara canis*) in a zoological garden per month (*N* = 156). The models were implemented in the R environment (14) using the “glmer” function, package lmerTest (15). Season in the respective study year (autumn 2012: October–November 2012; winter 2013: December 2012–February 2013; spring 2013: March–May 2013; summer 2013: June–August 2013; autumn 2013: September–November 2013), the number of samples the zoo contributed per month and anthelmintic treatment (yes/no) were included as fixed factors and zoo identity as a random effect. Because Taeniidae are transmitted to carnivores by ingestion of infected intermediate hosts, the fact whether the zoo fed game or meat from other wildlife (e.g., zoo-bred or roadkilled) was included as an explanatory variable for this parasite. Each full model was compared to a null model comprising only the random effect in a likelihood ratio test using the R-function ANOVA with the argument “test” set to “Chisq.” Statistical significance was inferred if the specific *P*-value of the factor as well as the *P*-value of the likelihood ratio test were ≤0.05. Multiple comparisons (Tukey contrasts) between all 5 levels of season (autumn 2012, winter 2012/2013, spring 2013, summer 2013, and autumn 2013) were performed using the function “glht,” package multcomp (16) with single-step *P*-value adjustment.

## Results

### Captive Wolf Management

Among the 14 participating zoological gardens, the study population comprised 72 European gray wolves, inhabiting 18 enclosures. Enclosure size ranged from 500 to 11,000 m^2^. Wolves lived in packs of 2–10 individuals, and the space available per wolf showed a large variation from 265 to 2,857 m^2^. Only two zoos kept wolves in mixed-species exhibits, together with two brown bears each. In all zoos, wolves were fed one to six times a week on meat originating from slaughterhouses (horse and/or beef). Furthermore, 11 zoos additionally fed wild boar and different deer species from roadkills, as well as excess animals from the zoo itself. In one zoo, wolves also received a small amount of fish. Dogs were allowed to enter all zoos; thus, the impact of this management factor on endoparasite infection of the captive wolves could not be evaluated statistically.

### Results of Coproscopical Examinations

In total, 1,041 wolf fecal samples were analyzed. The number of samples per zoo and month ranged from 0 to 25. In 26 cases, bulk samples (i.e., a single mixed sample per zoo and month) were collected. Only in March 2013, all 14 participating zoos contributed samples. In October 2013 and September 2013, 10 zoos contributed samples, and in the remaining study months, the number of contribution zoos was once again lower. Overall, 156 monthly data points (parasite presence/absence on zoo level) resulted from the study period of 14 months.

**Figure 1:**
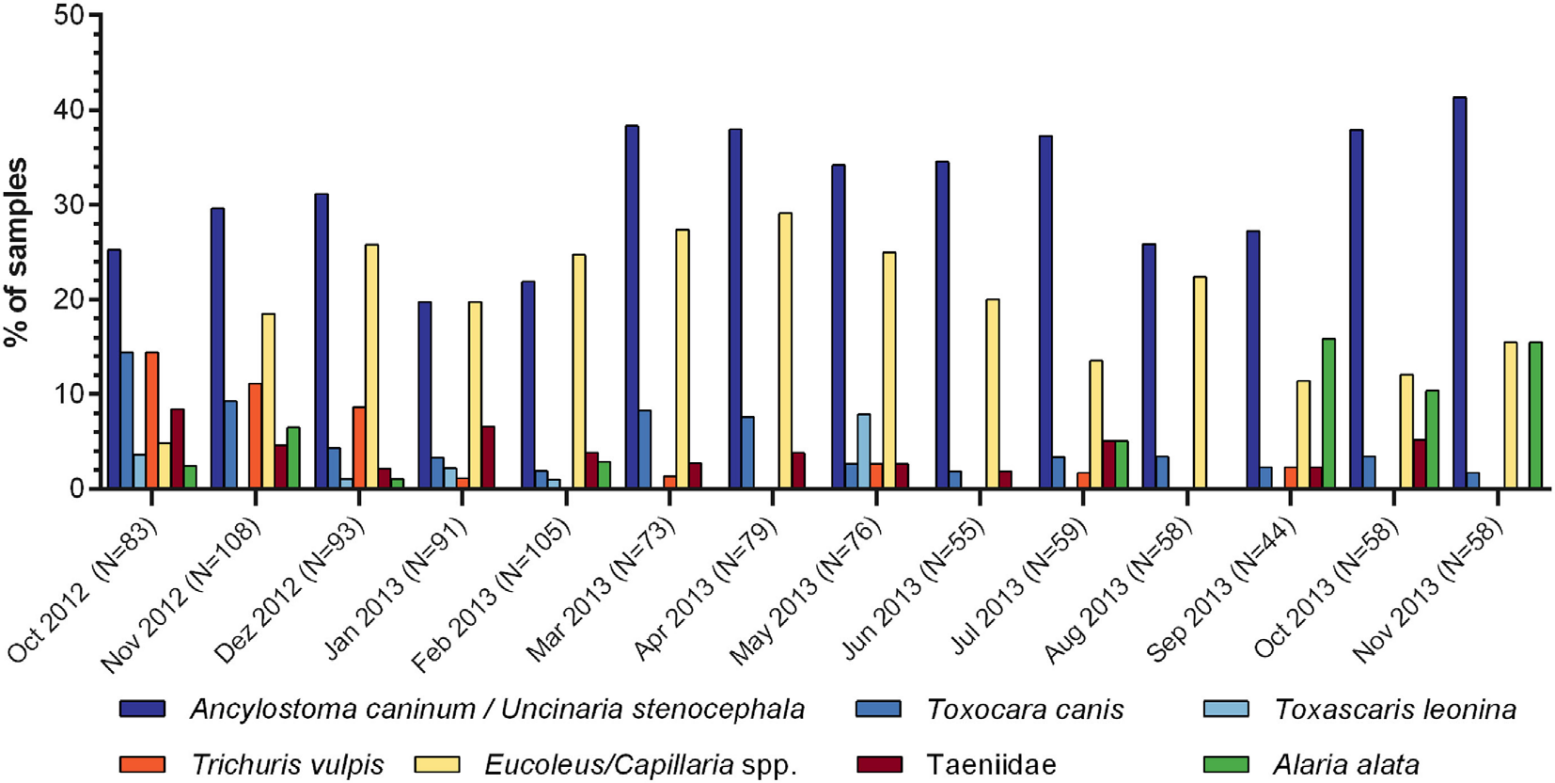
Monthly detection frequencies of Ancylostomatidae, *T. canis, T. leonina, T. vulpis, Capillaria/Eucoleus* spp., Taeniidae, and *A. alata* eggs in fecal samples from captive European gray wolves in Germany. Monthly sample size is indicated in brackets.

**Figure 2:**
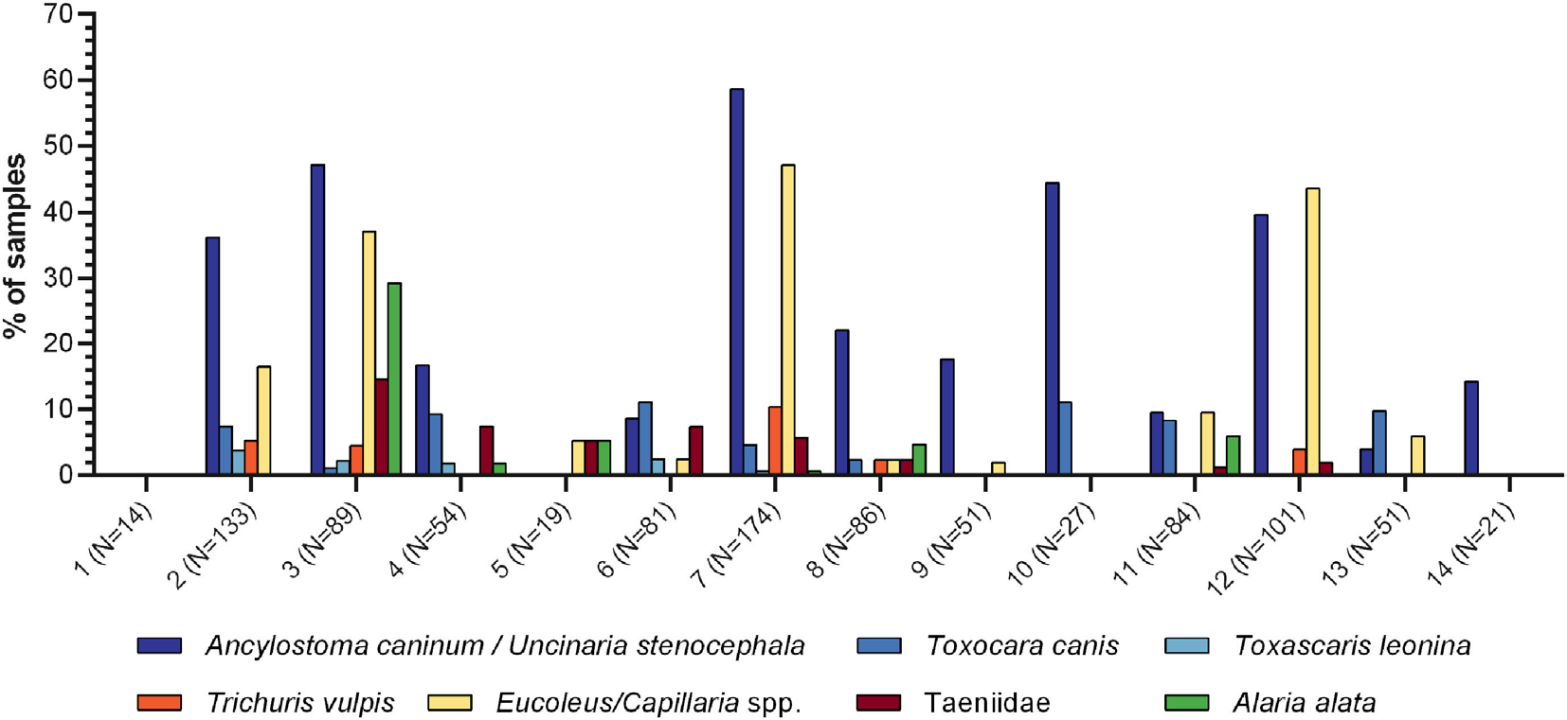
Detection frequencies of Ancylostomatidae, *T. canis, T. leonina, T. vulpis, Capillaria/Eucoleus* spp., Taeniidae, and *A. alata* eggs per individual zoo. The sample size for each of the fourteen zoos is indicated in brackets.

In total, 23 different parasite egg morphotypes were detected, some of which are considered as gastrointestinal passengers. Seven helminth egg morphotypes were detected with high frequency, including five nematodes (*Toxocara canis, Toxascaris leonina, Capillaria/Eucoleus* spp., *Trichuris vulpis*, and Ancylostomatidae [*Ancylostoma* or *Uncinaria* spp.]), one cestode (Taeniidae), and one trematode (*Alaria alata*). A percentage rate of 44.76 of all fecal samples was positive for at least one of these egg morphotypes. Overall, Ancylostomatidae showed the highest frequency (30.84% of all samples), followed by *Capillaria*/*Eucoleus* spp. (19.88%), *T. canis* (5.19%), taeniids (3.75%), *T. vulpis* and *A. alata* (3.65% each), and *T. leonina* (1.25%). Figure 1 shows monthly egg detection frequencies of these parasite eggs among all participating zoos, while Tables 1–7 list monthly egg detection frequencies and EPG ranges among the individual samples from each zoo. The monthly number of positive zoos is pictured in Figure S1 in Supplementary Material. The total egg detection rate for respective parasite eggs per zoo is shown in Figure 2. In addition to above mentioned egg morphotypes, eggs of *Spirocerca lupi* (0.58%), *Opisthorchis* spp. (0.19%), and *Diphyllobothrium latum* (0.10%) were detected. Furthermore, first-stage larvae of *Angiostrongulus vasorum* were identified; however, the detected frequency of 0.67% is certainly underestimated as the applied sedimentation-flotation method is not reliable for detection of lungworm larvae. Besides eggs of canine-associated parasites, a variety of eggs originating from food animals were found as gastrointestinal passengers: *Porrocaecum* spp. (2.59%), *Parascaris equorum* (0.86%), *Echinuria* spp. (0.38%), *Nematodirus* spp. (0.48%), *Heterakis* spp. (0.38%), *Moniezia* spp. (0.29%), *Ascaris suum* (0.19%), *Acanthocephalus* spp. (0.10%), and *Paramphistomum* spp. (0.10%). Furthermore, in 5.86% of the fecal samples, eggs of *Fasciola hepatica* were identified. However, it remains unknown whether the wolves, the food animals, or both served as the parasite host. Similarly, coccidian oocysts, which were present in 4.90% of the samples, could not be attributed to the wolves or food animals as they were unsporulated or could not be sufficiently differentiated at their sporulated stage due to freeze-thaw degradation.

**Table 1:** Monthly hookworm (Ancylostomatidae) egg detection frequency (number of positive samples/number of contributed samples) per zoo.

Zoo no.	October 2012	November 2012	December 2012	January 2013	February 2013	March 2013	April 2013	May 2013	June 2013	July 2013	August 2013	September 2013	October 2013	November 2013
1	0/2	0/2	0/1*	0/1*	0/1*	0/1*	0/1*	0/1*	0/1*	0/1*	0/1*	0/1*	0/1*	0/1*
	(0)	(0)	(0)	(0)	(0)	(0)	(0)	(0)	(0)	(0)	(0)	(0)	(0)	(0)
2	9/20	3/23	6/15	1/13	3/13	7/13	2/14	5/12	1/10	4/12	7/11	5/11	6/11	9/10
	(0–150)	(0–75)	(0–25)	(0)	(0–50)	(0–150)	(0–25)	(0–50)	(0)	(0–25)	(0–50)	(0–25)	(0)	(0–25)
3	n.a.	3/6	3/6	4/8	3/11	5/6	5/8	2/7	4/6	1/5	1/6	3/5	5/6	3/9
	(n.a.)	(100–200)	(0–25)	(0–100)	(0–50)	(0–125)	(0–175)	(25–100)	(0–100)	(50)	(175)	(0–350)	(0–100)	(0–25)
4	1/6	3/10	2/10	2/7	0/5	0/3	0/3	n.a.	n.a.	n.a.	1/3	n.a.	0/7	n.a.
	(0)	(25–50)	(25–100)	(0)	(0)	(0)	(0)	(n.a.)	(n.a.)	(n.a.)	(0)	(n.a.)	(0)	(n.a.)
5	0/5	0/3	n.a.	0/1*	0/1*	0/1*	0/1*	0/1*	0/1*	0/1*	0/1*	0/1*	0/1*	0/1*
	(0)	(0)	(n.a.)	(0)	(0)	(0)	(0)	(0)	(0)	(0)	(0)	(0)	(0)	(0)
6	1/9	2/12	1/13	0/11	0/11	n.a.	n.a.	0/7	0/3	n.a.	0/3	n.a.	1/6	2/6
	(0)	(0–25)	(0)	(0)	(0)	(n.a.)	(n.a.)	(0)	(0)	(n.a.)	(0)	(n.a.)	(0)	(0)
7	8/19	14/25	10/16	6/15	6/12	5/13	7/14	9/10	9/11	9/10	5/10	n.a.	7/10	7/9
	(0–275)	(0–325)	(0–75)	(0–75)	(0–75)	(25–50)	(0–275)	(0–225)	(0–600)	(0–150)	(0–50)	(n.a.)	(0–125)	(0–25)
8	2/7	3/9	4/9	1/8	1/10	1/7	2/10	2/10	1/3	1/7	n.a.	n.a.	n.a.	1/6
	(0–25)	(25–175)	(0–25)	(0)	(0)	(125)	(0–150)	(25–75)	(100)	(25)	(n.a.)	(n.a.)	(n.a.)	(0)
9	0/7	0/6	n.a.	0/4	0/5	n.a.	3/5	2/5	2/5	n.a.	1/4	0/3	1/4	0/3
	(0)	(0)	(n.a.)	(0)	(0)	(n.a.)	(0–25)	(0–25)	(0–175)	(n.a.)	(0)	(0)	(0)	(0)
10	0/3	0/2	1/2	1/2	0/2	1/2	2/2	0/2	2/2	2/2	n.a.	2/2	1/2	0/2
	(0)	(0)	(50)	(25)	(0)	(25)	(0)	(0)	(0–100)	(0–50)	(n.a.)	(0–25)	(0)	(0)
11	0/5	0/6	0/7	0/8	0/8	1/7	3/7	1/7	0/7	2/7	0/9	1/6	n.a.	n.a.
	(0)	(0)	(0)	(0)	(0)	(25)	(0–25)	(0)	(0)	(0–75)	(0)	(0)	(n.a.)	(n.a.)
12	n.a.	4/5	1/8	3/10	10/15	8/8	6/9	3/11	0/5	2/8	0/5	1/8	0/4	2/5
	(n.a.)	(0–675)	(0)	(0–25)	(0–125)	(0–125)	(0–275)	(0–100)	(0)	(0)	(0)	(25)	(0)	(0–25)
13	n.a.	n.a.	1/6	n.a.	0/9	0/10	0/3	0/2	n.a.	1/3	0/3	0/6	0/5	0/4
	(n.a.)	(n.a.)	(25)	(n.a.)	(0)	(0)	(0)	(0)	(n.a.)	(25)	(0)	(0)	(0)	(0)
14	n.a.	n.a.	n.a.	0/3	0/2	0/2	0/2	2/2	0/1*	0/3	0/2	0/1*	1/1*	0/2
	(n.a.)	(n.a.)	(n.a.)	(0)	(0)	(0)	(0)	(0)	(0)	(0)	(0)	(0)	(0)	(0)

*The range of eggs per gram feces as determined by the McMaster method is indicated in brackets*.

Bulk samples are marked with an asterisk (*).

**Table 2:** Monthly *Toxocara canis* egg detection frequency (number of positive samples/number of contributed samples) per zoo.

Zoo no.	October 2012	November 2012	December 2012	January 2013	February 2013	March 2013	April 2013	May 2013	June 2013	July 2013	August 2013	September 2013	October 2013	November 2013
1	0/2	0/2	0/1*	0/1*	0/1*	0/1*	0/1*	0/1*	0/1*	0/1*	0/1*	0/1*	0/1*	0/1*
	(0)	(0)	(0)	(0)	(0)	(0)	(0)	(0)	(0)	(0)	(0)	(0)	(0)	(0)
2	3/20	3/23	1/15	0/13	1/13	3/13	2/14	1/12	0/10	0/12	0/11	0/11	0/11	0/10
	(0–25)	(0)	(0)	(0)	(0)	(0)	(0)	(0)	(0)	(0)	(0)	(0)	(0)	(0)
3	n.a.	0/6	0/6	0/8	0/11	0/6	1/8	0/7	0/6	0/5	0/6	0/5	0/6	0/9
	(n.a.)	(0)	(0)	(0)	(0)	(0)	(0)	(0)	(0)	(0)	(0)	(0)	(0)	(0)
4	0/6	1/10	1/10	0/7	0/5	1/3	2/3	n.a.	n.a.	n.a.	0/3	n.a.	0/7	n.a.
	(0)	(0)	(0)	(0)	(0)	(0)	(0)	(n.a.)	(n.a.)	(n.a.)	(0)	(n.a.)	(0)	(n.a.)
5	0/5	0/3	n.a.	0/1*	0/1*	0/1*	0/1*	0/1*	0/1*	0/1*	0/1*	0/1*	0/1*	0/1*
	(0)	(0)	(n.a.)	(0)	(0)	(0)	(0)	(0)	(0)	(0)	(0)	(0)	(0)	(0)
6	6/9	2/12	1/13	0/11	0/11	n.a.	n.a.	0/7	0/3	n.a.	0/3	n.a.	0/6	0/6
	(0)	0	(0)	(0)	(0)	(n.a.)	(n.a.)	(0)	(0)	(n.a.)	(0)	(n.a.)	(0)	(0)
7	2/19	2/25	0/16	2/15	0/12	0/13	0/14	0/10	0/11	0/10	0/10	n.a.	1/10	1/9
	(0–50)	(0)	(0)	(0)	(0)	(0)	(0)	(0)	(0)	(0)	(0)	(n.a.)	(75)	(0)
8	0/7	1/9	0/9	0/8	0/10	0/7	0/10	0/10	1/3	0/7	n.a.	n.a.	n.a.	0/6
	(0)	(0)	(0)	(0)	(0)	(0)	(0)	(0)	(0)	(0)	(n.a.)	(n.a.)	(n.a.)	(0)
9	0/7	0/6	n.a.	0/4	0/5	n.a.	0/5	0/5	0/5	n.a.	0/4	0/3	0/4	0/3
	(0)	(0)	(n.a.)	(0)	(0)	(n.a.)	(0)	(0)	(0)	(n.a.)	(0)	(0)	(0)	(0)
10	0/3	1/2	0/2	1/2	0/2	0/2	1/2	0/2	0/2	0/2	n.a.	0/2	0/2	0/2
	(0)	(25)	(0)	(0)	(0)	(0)	(0)	(0)	(0)	(0)	(n.a.)	(0)	(0)	(0)
11	1/5	0/6	1/7	0/8	1/8	2/7	0/7	1/7	0/7	0/7	0/9	1/6	n.a.	n.a.
	(0)	(0)	(0)	(0)	(0)	(25–50)	(0)	(25)	(0)	(0)	(0)	(0)	(n.a.)	(n.a.)
12	n.a.	0/5	0/8	0/10	0/15	0/8	0/9	0/11	0/5	0/8	0/5	0/8	0/4	0/5
	(n.a.)	(0)	(0)	(0)	(0)	(0)	(0)	(0)	(0)	(0)	(0)	(0)	(0)	(0)
13	n.a.	n.a.	0/6	n.a.	0/9	0/10	0/3	0/2	n.a.	2/3	2/3	0/6	1/5	0/4
	(n.a.)	(n.a.)	(0)	(n.a.)	(0)	(0)	(0)	(0)	(n.a.)	(25–525)	(25–50)	(0)	(0)	(0)
14	n.a.	n.a.	n.a.	0/3	0/2	0/2	0/2	0/2	0/1*	0/3	0/2	0/1*	0/1*	0/2
	(n.a.)	(n.a.)	(n.a.)	(0)	(0)	(0)	(0)	(0)	(0)	(0)	(0)	(0)	(0)	(0)

*The range of eggs per gram feces as determined by the McMaster method is indicated in brackets*.

Bulk samples are marked with an asterisk (*).

**Table 3:** Monthly *Toxascaris leonina* egg detection frequency (number of positive samples/number of contributed samples) per zoo.

Zoo no.	October 2012	November 2012	December 2012	January 2013	February 2013	March 2013	April 2013	May 2013	June 2013	July 2013	August 2013	September 2013	October 2013	November 2013
1	0/2	0/2	0/1*	0/1*	0/1*	0/1*	0/1*	0/1*	0/1*	0/1*	0/1*	0/1*	0/1*	0/1*
	(0)	(0)	(0)	(0)	(0)	(0)	(0)	(0)	(0)	(0)	(0)	(0)	(0)	(0)
2	0/20	0/23	0/15	0/13	1/13	0/13	0/14	6/12	0/10	0/12	0/11	0/11	0/11	0/10
	(0)	(0)	(0)	(0)	(0)	(0)	(0)	(0–75)	(0)	(0)	(0)	(0)	(0)	(0)
3	n.a.	0/6	0/6	2/8	0/11	0/6	0/8	0/7	0/6	0/5	0/6	0/5	0/6	0/9
	(n.a.)	(0)	(0)	(0)	(0)	(0)	(0)	(0)	(0)	(0)	(0)	(0)	(0)	(0)
4	1/6	0/10	0/10	0/7	0/5	0/3	0/3	n.a.	n.a.	n.a.	0/3	n.a.	0/7	n.a.
	(0)	(0)	(0)	(0)	(0)	(0)	(0)	(n.a.)	(n.a.)	(n.a.)	(0)	(n.a.)	(0)	(n.a.)
5	0/5	0/3	n.a.	0/1*	0/1*	0/1*	0/1*	0/1*	0/1*	0/1*	0/1*	0/1*	0/1*	0/1*
	(0)	(0)	(n.a.)	(0)	(0)	(0)	(0)	(0)	(0)	(0)	(0)	(0)	(0)	(0)
6	2/9	0/12	0/13	0/11	0/11	n.a.	n.a.	0/7	0/3	n.a.	0/3	n.a.	0/6	0/6
	(0)	(0)	(0)	(0)	(0)	(n.a.)	(n.a.)	(0)	(0)	(n.a.)	(0)	(n.a.)	(0)	(0)
7	0/19	0/25	1/16	0/15	0/12	0/13	0/14	0/10	0/11	0/10	0/10	n.a.	0/10	0/9
	(0)	(0)	(0)	(0)	(0)	(0)	(0)	(0)	(0)	(0)	(0)	(n.a.)	(0)	(0)
8	0/7	0/9	0/9	0/8	0/10	0/7	0/10	0/10	0/3	0/7	n.a.	n.a.	n.a.	0/6
	(0)	(0)	(0)	(0)	(0)	(0)	(0)	(0)	(0)	(0)	(n.a.)	(n.a.)	(n.a.)	(0)
9	0/7	0/6	n.a.	0/4	0/5	n.a.	0/5	0/5	0/5	n.a.	0/4	0/3	0/4	0/3
	(0)	(0)	(n.a.)	(0)	(0)	(n.a.)	(0)	(0)	(0)	(n.a.)	(0)	(0)	(0)	(0)
10	0/3	0/2	0/2	0/2	0/2	0/2	0/2	0/2	0/2	0/2	n.a.	0/2	0/2	0/2
	(0)	(0)	(0)	(0)	(0)	(0)	(0)	(0)	(0)	(0)	(n.a.)	(0)	(0)	(0)
11	0/5	0/6	0/7	0/8	0/8	0/7	0/7	0/7	0/7	0/7	0/9	0/6	n.a.	n.a.
	(0)	(0)	(0)	(0)	(0)	(0)	(0)	(0)	(0)	(0)	(0)	(0)	(n.a.)	(n.a.)
12	n.a.	0/5	0/8	0/10	0/15	0/8	0/9	0/11	0/5	0/8	0/5	0/8	0/4	0/5
	(n.a.)	(0)	(0)	(0)	(0)	(0)	(0)	(0)	(0)	(0)	(0)	(0)	(0)	(0)
13	n.a.	n.a.	0/6	n.a.	0/9	0/10	0/3	0/2	n.a.	0/3	0/3	0/6	0/5	0/4
	(n.a.)	(n)	(0)	(n.a.)	(0)	(0)	(0)	(0)	(n.a.)	(0)	(0)	(0)	(0)	(0)
14	n.a.	n.a.	n.a.	0/3	0/2	0/2	0/2	0/2	0/1*	0/3	0/2	0/1*	0/1*	0/2
	(n.a.)	(n.a.)	(n.a.)	(0)	(0)	(0)	(0)	(0)	(0)	(0)	(0)	(0)	(0)	(0)

*The range of eggs per gram feces as determined by the McMaster method is indicated in brackets*.

Bulk samples are marked with an asterisk (*).

**Table 4:** Monthly *Trichuris vulpis* egg detection frequency (number of positive samples/number of contributed samples) per zoo.

Zoo no.	October 2012	November 2012	December 2012	January 2013	February 2013	March 2013	April 2013	May 2013	June 2013	July 2013	August 2013	September 2013	October 2013	November 2013
1	0/2	0/2	0/1*	0/1*	0/1*	0/1*	0/1*	0/1*	0/1*	0/1*	0/1*	0/1*	0/1*	0/1*
	(0)	(0)	(0)	(0)	(0)	(0)	(0)	(0)	(0)	(0)	(0)	(0)	(0)	(0)
2	0/20	8/23	2/15	0/13	0/13	0/13	0/14	0/12	0/10	0/12	0/11	0/11	0/11	0/10
	(0)	(0)	(0)	(0)	(0)	(0)	(0)	(0)	(0)	(0)	(0)	(0)	(0)	(0)
3	n.a.	3/6	0/6	0/8	0/11	0/6	0/8	1/7	0/6	0/5	0/6	0/5	0/6	0/9
	(n.a.)	(250–775)	(0)	(0)	(0)	(0)	(0)	(25)	(0)	(0)	(0)	(0)	(0)	(0)
4	0/6	0/10	0/10	0/7	0/5	0/3	0/3	n.a.	n.a.	n.a.	0/3	n.a.	0/7	n.a.
	(0)	(0)	(0)	(0)	(0)	(0)	(0)	(n.a.)	(n.a.)	(n.a.)	(0)	(n.a.)	(0)	(n.a.)
5	0/5	0/3	n.a.	0/1*	0/1*	0/1*	0/1*	0/1*	0/1*	0/1*	0/1*	0/1*	0/1*	0/1*
	(0)	(0)	(n.a.)	(0)	(0)	(0)	(0)	(0)	(0)	(0)	(0)	(0)	(0)	(0)
6	0/9	0/12	0/13	0/11	0/11	n.a.	n.a.	0/7	0/3	n.a.	0/3	n.a.	0/6	0/6
	(0)	(0)	(0)	(0)	(0)	(n.a.)	(n.a.)	(0)	(0)	(n.a.)	(0)	(n.a.)	(0)	(0)
7	10/19	0/25	5/16	1/15	0/12	0/13	0/14	1/10	0/11	1/10	0/10	n.a.	0/10	0/9
	(25–425)	(0)	(0–50)	(25)	(0)	(0)	(0)	(50)	(0)	(0)	(0)	(n.a.)	(0)	(0)
8	2/7	0/9	0/9	0/8	0/10	0/7	0/10	0/10	0/3	0/7	n.a.	n.a.	n.a.	0/6
	(0–25)	(0)	(0)	(0)	(0)	(0)	(0)	(0)	(0)	(0)	(n.a.)	(n.a.)	(n.a.)	(0)
9	0/7	0/6	n.a.	0/4	0/5	n.a.	0/5	0/5	0/5	n.a.	0/4	0/3	0/4	0/3
	(0)	(0)	(n.a.)	(0)	(0)	(n.a.)	(0)	(0)	(0)	(n.a.)	(0)	(0)	(0)	(0)
10	0/3	0/2	0/2	0/2	0/2	0/2	0/2	0/2	0/2	0/2	n.a.	0/2	0/2	0/2
	(0)	(0)	(0)	(0)	(0)	(0)	(0)	(0)	(0)	(0)	(n.a.)	(0)	(0)	(0)
11	0/5	0/6	0/7	0/8	0/8	0/7	0/7	0/7	0/7	0/7	0/9	0/6	n.a.	n.a.
	(0)	(0)	(0)	(0)	(0)	(0)	(0)	(0)	(0)	(0)	(0)	(0)	(n.a.)	(n.a.)
12	n.a.	1/5	1/8	0/10	0/15	1/8	0/9	0/11	0/5	0/8	0/5	1/8	0/4	0/5
	(n.a.)	(0)	(0)	(0)	(0)	(75)	(0)	(0)	(0)	(0)	(0)	(25)	(0)	(0)
13	n.a.	n.a.	0/6	n.a.	0/9	0/10	0/3	0/2	n.a.	0/3	0/3	0/6	0/5	0/4
	(n.a.)	(n)	(0)	(n.a.)	(0)	(0)	(0)	(0)	(n.a.)	(0)	(0)	(0)	(0)	(0)
14	n.a.	n.a.	n.a.	0/3	0/2	0/2	0/2	0/2	0/1*	0/3	0/2	0/1*	0/1*	0/2
	(n.a.)	(n.a.)	(n.a.)	(0)	(0)	(0)	(0)	(0)	(0)	(0)	(0)	(0)	(0)	(0)

*The range of eggs per gram feces as determined by the McMaster method is indicated in brackets*.

Bulk samples are marked with an asterisk (*).

**Table 5:** Monthly *Capillaria/Eucoleus* spp. egg detection frequency (number of positive samples/number of contributed samples) per zoo.

Zoo no.	October 2012	November 2012	December 2012	January 2013	February 2013	March 2013	April 2013	May 2013	June 2013	July 2013	August 2013	September 2013	October 2013	November 2013
1	0/2	0/2	0/1*	0/1*	0/1*	0/1*	0/1*	0/1*	0/1*	0/1*	0/1*	0/1*	0/1*	0/1*
	(0)	(0)	(0)	(0)	(0)	(0)	(0)	(0)	(0)	(0)	(0)	(0)	(0)	(0)
2	1/20	0/23	0/15	3/13	4/13	8/13	3/14	3/12	3/10	1/12	1/11	0/11	2/11	2/10
	(0)	(0)	(0)	(0)	(0–25)	(0–475)	(0–50)	(0)	(0–75)	(75)	(25)	(0)	(0)	(0)
3	n.a.	2/6	4/6	4/8	3/11	1/6	4/8	3/7	1/6	2/5	2/6	3/5	3/6	1/9
	(n.a.)	(0–75)	(0–275)	(275–6,650)	(400–3,550)	(25)	(0–925)	(0–1,350)	(100)	(0–50)	(25–2,175)	(25–2,275)	(25)	(25)
4	0/6	0/10	0/10	0/7	0/5	0/3	0/3	n.a.	n.a.	n.a.	0/3	n.a.	0/7	n.a.
	(0)	(0)	(0)	(0)	(0)	(0)	(0)	(n.a.)	(n.a.)	(n.a.)	(0)	(n.a.)	(0)	(n.a.)
5	0/5	1/3	n.a.	0/1*	0/1*	0/1*	0/1*	0/1*	0/1*	0/1*	0/1Ü	0/1*	0/1*	0/1*
	(0)	(0)	(n.a.)	(0)	(0)	(0)	(0)	(0)	(0)	(0)	(0)	(0)	(0)	(0)
6	1/9	1/12	0/13	0/11	0/11	n.a.	n.a.	0/7	0/3	n.a.	0/3	n.a.	0/6	0/6
	(0)	(0)	(0)	(0)	(0)	(n.a.)	(n.a.)	(0)	(0)	(n.a.)	(0)	(n.a.)	(0)	(0)
7	0/19	12/25	15/16	8/15	9/12	4/13	9/14	7/10	5/11	1/10	6/10	n.a.	2/10	4/9
	(0)	(0–325)	(0–125)	(0–75)	(0–100)	(0)	(0–300)	(0–275)	(0–150)	(75)	(0–200)	(n.a.)	(25–100)	(0–100)
8	1/7	0/9	0/9	0/8	0/10	0/7	0/10	0/10	1/3	0/7	n.a.	n.a.	n.a.	0/6
	(0)	(0)	(0)	(0)	(0)	(0)	(0)	(0)	(550)	(0)	(n.a.)	(n.a.)	(n.a.)	(0)
9	0/7	0/6	n.a.	0/4	0/5	n.a.	0/5	0/5	0/5	n.a.	0/4	1/3	0/4	0/3
	(0)	(0)	(n.a.)	(0)	(0)	(n.a.)	(0)	(0)	(0)	(n.a.)	(0)	(0)	(0)	(0)
10	0/3	0/2	0/2	0/2	0/2	0/2	0/2	0/2	0/2	0/2	n.a.	0/2	0/2	0/2
	(0)	(0)	(0)	(0)	(0)	(0)	(0)	(0)	(0)	(0)	(n.a.)	(0)	(0)	(0)
11	1/5	0/6	0/7	0/8	1/8	0/7	2/7	0/7	0/7	1/7	2/9	1/6	n.a.	n.a.
	(0)	(0)	(0)	(0)	(0)	(0)	(0)	(0)	(0)	(75)	(0–25)	(0)	(n.a.)	(n.a.)
12	n.a.	4/5	3/8	3/10	9/15	6/8	5/9	6/11	1/5	3/8	2/5	0/8	0/4	2/5
	(n.a.)	(50–475)	(0–50)	(0–125)	(0–75)	(0–125)	(0–200)	(0–25)	(50)	(0–100)	(0)	(0)	(0)	(0–25)
13	n.a.	n.a.	2/6	n.a.	0/9	1/10	0/3	0/2	n.a.	0/3	0/3	0/6	0/5	0/4
	(n.a.)	(n.a.)	(25–50)	(n.a.)	(0)	(0)	(0)	(0)	(n.a.)	(0)	(0)	(0)	(0)	(0)
14	n.a.	n.a.	n.a.	0/3	0/2	0/2	0/2	0/2	0/1*	0/3	0/2	0/1*	0/1*	0/2
	(n.a.)	(n.a.)	(n.a.)	(0)	(0)	(0)	(0)	(0)	(0)	(0)	(0)	(0)	(0)	(0)

*The range of eggs per gram feces as determined by the McMaster method is indicated in brackets*.

Bulk samples are marked with an asterisk (*).

**Table 6:** Monthly taeniid egg detection frequency (number of positive samples/number of contributed samples) per zoo.

Zoo no.	October 2012	November 2012	December 2012	January 2013	February 2013	March 2013	April 2013	May 2013	June 2013	July 2013	August 2013	September 2013	October 2013	November 2013
1	0/2	0/2	0/1*	0/1*	0/1*	0/1*	0/1*	0/1*	0/1*	0/1*	0/1*	0/1*	0/1*	0/1*
	(0)	(0)	(0)	(0)	(0)	(0)	(0)	(0)	(0)	(0)	(0)	(0)	(0)	(0)
2	0/20	0/23	0/15	0/13	0/13	0/13	0/14	0/12	0/10	0/12	0/11	0/11	0/11	0/10
	(0)	(0)	(0)	(0)	(0)	(0)	(0)	(0)	(0)	(0)	(0)	(0)	(0)	(0)
3	n.a.	3/6	1/6	0/8	0/11	1/6	1/8	2/7	1/6	2/5	0/6	0/5	2/6	0/9
	(n.a.)	(0–50)	(25)	(0)	(0)	(0)	(25)	(0–200)	(0)	(0)	(0)	(0)	(0–7,750)	(0)
4	0/6	1/10	0/10	0/7	2/5	0/3	1/3	n.a.	n.a.	n.a.	0/3	n.a.	0/7	n.a.
	(0)	(0)	(0)	(0)	(0–275)	(0)	(0)	(n.a.)	(n.a.)	(n.a.)	(0)	(n.a.)	(0)	(n.a.)
5	0/5	0/3	n.a.	0/1*	0/1*	0/1*	0/1*	0/1*	0/1*	0/1*	0/1*	1/1*	0/1*	0/1*
	(0)	(0)	(n.a.)	(0)	(0)	(0)	(0)	(0)	(0)	(0)	(0)	(325)	(0)	(0)
6	5/9	0/12	0/13	0/11	0/11	n.a.	n.a.	0/7	0/3	n.a.	0/3	n.a.	1/6	0/6
	(0)	(0)	(0)	(0)	(0)	(n.a.)	(n.a.)	(0)	(0)	(n.a.)	(0)	(n.a.)	(0)	(0)
7	2/19	1/25	1/16	6/15	0/12	0/13	0/14	0/10	0/11	0/10	0/10	n.a.	0/10	0/9
	(0)	(0)	(0)	(0)	(0)	(0)	(0)	(0)	(0)	(0)	(0)	(n.a.)	(0)	(0)
8	0/7	0/9	0/9	0/8	0/10	1/7	1/10	0/10	0/3	0/7	n.a.	n.a.	n.a.	0/6
	(0)	(0)	(0)	(0)	(0)	(25)	(0)	(0)	(0)	(0)	(n.a.)	(n.a.)	(n.a.)	(0)
9	0/7	0/6	n.a.	0/4	0/5	n.a.	0/5	0/5	0/5	n.a.	0/4	0/3	0/4	0/3
	(0)	(0)	(n.a.)	(0)	(0)	(n.a.)	(0)	(0)	(0)	(n.a.)	(0)	(0)	(0)	(0)
10	0/3	0/2	0/2	0/2	0/2	0/2	0/2	0/2	0/2	0/2	n.a.	0/2	0/2	0/2
	(0)	(0)	(0)	(0)	(0)	(0)	(0)	(0)	(0)	(0)	(n.a.)	(0)	(0)	(0)
11	0/5	0/6	0/7	0/8	0/8	0/7	0/7	0/7	0/7	1/7	0/9	0/6	n.a.	n.a.
	(0)	(0)	(0)	(0)	(0)	(0)	(0)	(0)	(0)	(0)	(0)	(0)	(n.a.)	(n.a.)
12	n.a.	0/5	0/8	0/10	2/15	0/8	0/9	0/11	0/5	0/8	0/5	0/8	0/4	0/5
	(n.a.)	(0)	(0)	(0)	(0)	(0)	(0)	(0)	(0)	(0)	(0)	(0)	(0)	(0)
13	n.a.	n.a.	0/6	n.a.	0/9	0/10	0/3	0/2	n.a.	0/3	0/3	0/6	0/5	0/4
	(n.a.)	(n)	(0)	(n.a.)	(0)	(0)	(0)	(0)	(n.a.)	(0)	(0)	(0)	(0)	(0)
14	n.a.	n.a.	n.a.	0/3	0/2	0/2	0/2	0/2	0/1*	0/3	0/2	0/1*	0/1*	0/2
	(n.a.)	(n)	(n.a.)	(0)	(0)	(0)	(0)	(0)	(0)	(0)	(0)	(0)	(0)	(0)

*The range of eggs per gram feces as determined by the McMaster method is indicated in brackets*.

Bulk samples are marked with an asterisk (*).

**Table 7:** Monthly *Alaria alata* egg detection frequency (number of positive samples/number of contributed samples) per zoo.

Zoo no.	October 2012	November 2012	December 2012	January 2013	February 2013	March 2013	April 2013	May 2013	June 2013	July 2013	August 2013	September 2013	October 2013	November 2013
1	0/2	0/2	0/1*	0/1*	0/1*	0/1*	0/1*	0/1*	0/1*	0/1*	0/1*	0/1*	0/1*	0/1*
	(0)	(0)	(0)	(0)	(0)	(0)	(0)	(0)	(0)	(0)	(0)	(0)	(0)	(0)
2	0/20	0/23	0/15	0/13	0/13	0/13	0/14	0/12	0/10	0/12	0/11	0/11	0/11	0/10
	(0)	(0)	(0)	(0)	(0)	(0)	(0)	(0)	(0)	(0)	(0)	(0)	(0)	(0)
3	n.a.	5/6	0/6	0/8	1/11	0/6	0/8	0/7	0/6	2/5	0/6	4/5	6/6	8/9
	(n.a.)	(0)	(0)	(0)	(0)	(0)	(0)	(0)	(0)	(0)	(0)	(0)	(0–75)	(0–75)
4	0/6	0/10	0/10	0/7	1/5	0/3	0/3	n.a.	n.a.	n.a.	0/3	n.a.	0/7	n.a.
	(0)	(0)	(0)	(0)	(0)	(0)	(0)	(n.a.)	(n.a.)	(n.a.)	(0)	(n.a.)	(0)	(n.a.)
5	1/5	0/3	n.a.	0/1*	0/1*	0/1*	0/1*	0/1*	0/1*	0/1*	0/1*	0/1*	0/1*	0/1*
	(0)	(0)	(n.a.)	(0)	(0)	(0)	(0)	(0)	(0)	(0)	(0)	(0)	(0)	(0)
6	0/9	0/12	0/13	0/11	0/11	n.a.	n.a.	0/7	0/3	n.a.	0/3	n.a.	0/6	0/6
	(0)	(0)	(0)	(0)	(0)	(n.a.)	(n.a.)	(0)	(0)	(n.a.)	(0)	(n.a.)	(0)	(0)
7	1/19	0/25	0/16	0/15	0/12	0/13	0/14	0/10	0/11	0/10	0/10	n.a.	0/10	0/9
	(0)	(0)	(0)	(0)	(0)	(0)	(0)	(0)	(0)	(0)	(0)	(n.a.)	(0)	(0)
8	0/7	2/9	1/9	0/8	0/10	0/7	0/10	0/10	0/3	0/7	n.a.	n.a.	n.a.	1/6
	(0)	(0)	(0)	(0)	(0)	(0)	(0)	(0)	(0)	(0)	(n.a.)	(n.a.)	(n.a.)	(0)
9	0/7	0/6	n.a.	0/4	0/5	n.a.	0/5	0/5	0/5	n.a.	0/4	0/3	0/4	0/3
	(0)	(0)	(n.a.)	(0)	(0)	(n.a.)	(0)	(0)	(0)	(n.a.)	(0)	(0)	(0)	(0)
10	0/3	0/2	0/2	0/2	0/2	0/2	0/2	0/2	0/2	0/2	n.a.	0/2	0/2	0/2
	(0)	(0)	(0)	(0)	(0)	(0)	(0)	(0)	(0)	(0)	(n.a.)	(0)	(0)	(0)
11	0/5	0/6	0/7	0/8	1/8	0/7	0/7	0/7	0/7	1/7	0/9	3/6	n.a.	n.a.
	(0)	(0)	(0)	(0)	(0)	(0)	(0)	(0)	(0)	(0)	(0)	(0)	(n.a.)	(n.a.)
12	n.a.	0/5	0/8	0/10	0/15	0/8	0/9	0/11	0/5	0/8	0/5	0/8	0/4	0/5
	(n.a.)	(0)	(0)	(0)	(0)	(0)	(0)	(0)	(0)	(0)	(0)	(0)	(0)	(0)
13	n.a.	n.a.	0/6	n.a.	0/9	0/10	0/3	0/2	n.a.	0/3	0/3	0/6	0/5	0/4
	(n.a.)	(n.a.)	(0)	(n.a.)	(0)	(0)	(0)	(0)	(n.a.)	(0)	(0)	(0)	(0)	(0)
14	n.a.	n.a.	n.a.	0/3	0/2	0/2	0/2	0/2	0/1*	0/3	0/2	0/1*	0/1*	0/2
	(n.a.)	(n.a.)	(n.a.)	(0)	(0)	(0)	(0)	(0)	(0)	(0)	(0)	(0)	(0)	(0)

*The range of eggs per gram feces as determined by the McMaster method is indicated in brackets*.

Bulk samples are marked with an asterisk (*).

### Results of Statistical Analyses

The effect of season and management factors was only assessed statistically for the four most prevalent parasites, namely Ancylostomatidae, *Capillaria/Eucoleus* spp., Taeniidae, and *T. canis*. Anthelmintic treatment negatively affected the probability of Ancylostomatidae egg detection (Table 8), whereas no statistically significant effects of management factors on the other parasites were found (Tables 9–11). Similarly, statistical analyses did not result in significant differences between seasons for any of these parasites (Tables 10–13). However, an increasing probability of *Toxocara canis* and Ancylostomatidae egg sdetection with increasing monthly sample size per zoo was observed (Tables 8 and 9).

**Table 8:** Results of generalized linear-mixed model testing the influence of different predictor variables on the probability of being infected with Ancylostomatidae.

Term	Estimate	SE	*z*	*P*
Intercept	−3.1528	1.0817	−2.915	**0.00356**
Winter 2013	−0.3457	0.8512	−0.406	0.68468
Spring 2013	1.8095	0.8916	2.030	**0.04240**
Summer 2013	1.1719	0.9258	1.266	0.20558
Autumn 2013	1.6651	0.8421	1.977	**0.04800**
Number of samples	0.7052	0.1399	5.042	**4.61e−07**
Anthelmintic treatment	−1.9807	0.9390	−2.109	**0.03490**

Significant P-values are shown in bold. Likelihood ratio test comparing the full model to a null model containing only the random effect: x^2^ = 32.966, df = 6, P = **1.064e−0.5.**

**Table 9:** Results of generalized linear-mixed model testing the influence of different predictor variables on *Toxocara canis* infections.

Term	Estimate	SE	*z*	*P*
Intercept	−2.33985	1.02538	−2.282	**0.0225**
Winter 2013	−1.29785	0.72940	−1.779	0.0752
Spring 2013	−0.60190	0.71860	−0.838	0.4023
Summer 2013	−2.70262	1.23124	−2.195	**0.0282**
Autumn 2013	−2.07825	0.87901	−2.364	**0.0181**
Number of samples	0.19986	0.08561	2.335	**0.0196**
Anthelmintic treatment	0.41279	0.94522	0.437	0.6623

*Significant P-values are shown in bold. Likelihood ratio test comparing the full model to a null model containing only the random effect: x^2^ = 24.952, df = 6, P = **0.0003486***.

**Table 10:** Results of generalized linear-mixed model testing of different predictor variables on the probability of *Capillaria/Eucoleus* spp. infections.

Term	Estimate	SE	*z*	*P*
Intercept	−3.29812	1.88567	−1.749	0.0803
Winter 2013	−1.22919	0.89753	−1.369	0.1708
Spring 2013	−0.82391	0.94777	−0.869	0.3847
Summer 2013	−0.18422	1.09981	−0.168	0.8670
Autumn 2013	0.99429	1.04862	−0.984	0.3430
Number of samples	−0.01123	0.14269	−0.079	0.9372
Anthelmintic treatment	3.86998	2.08820	1.853	0.0638

*Likelihood ratio test comparing the full model to a null model containing only the random effect: x^2^ = 6.5639, df = 6, P = 0.3631*.

**Table 11:** Results of generalized linear-mixed model testing the effect of different predictor variables on taeniid infections.

Term	Estimate	SE	*z*	*P*
Intercept	−5.58920	1.99841	−2.797	**0.0052**
Winter 2013	−0.77179	0.83946	−0.919	0.3580
Spring 2013	−0.05685	0.86827	−0.066	0.9478
Summer 2013	−0.16272	1.05442	−0.154	0.8774
Autumn 2013	−1.20535	1.00961	−1.194	0.2325
Number of samples	0.12855	0.09155	1.404	0.1603
Anthelmintic treatment	1.57436	1.25347	1.256	0.2091
Feeding of game	1.87909	1.49423	1.258	0.2086

*Significant P-values are shown in bold. Likelihood ratio test comparing the full model to a null model containing only the random effect: x^2^ = 12.584, df = 7, P = 0.08292*.

**Table 12:** Results of multiple comparisons between seasons regarding hookworm (Ancylostomatidae) infections.

Term	Estimate	SE	*z*	*P*
Winter 2013–autumn 2012	−0.3457	0.8512	−0.406	0.9942
Spring 2013–autumn 2012	1.8095	0.8916	2.030	0.2498
Summer 2013–autumn 2012	1.1719	0.9258	1.266	0.7103
Autumn 2013–autumn 2012	1.6651	0.8421	1.977	0.2750
Spring 2013–winter 2013	2.1552	0.8332	2.587	0.0719
Summer 2013–winter 2013	1.5176	0.8621	1.760	0.3946
Autumn 2013–winter 2013	2.0108	0.7908	2.543	0.0806
Summer 2013–spring 2013	−0.6376	0.8435	−0.756	0.9424
Autumn 2013–spring 2013	−0.1445	0.7190	−0.201	0.9996
Autumn 2013–summer 2013	0.4932	0.7834	0.630	0.9700

*P-values have been adjusted according to the single-step method using the function glht from the package multcomp in R*.

**Table 13:** Results of multiple comparisons between seasons regarding *Toxocara canis* infections.

Term	Estimate	SE	*z*	*P*
Winter 2013–autumn 2012	−1.2978	0.7294	−1.779	0.373
Spring 2013–autumn 2012	−0.6019	0.7186	−0.838	0.915
Summer 2013–autumn 2012	−2.7026	1.2312	−2.195	0.173
Autumn 2013–autumn 2012	−2.0782	0.8790	−2.364	0.119
Spring 2013–winter 2013	0.6959	0.6618	1.052	0.824
Summer 2013–winter 2013	−1.4048	1.1872	−1.183	0.752
Autumn 2013–winter 2013	−0.7804	0.8230	−0.948	0.872
Summer 2013–spring 2013	−2.1007	1.1713	−1.793	0.365
Autumn 2013–spring 2013	−1.4763	0.7970	−1.852	0.331
Autumn 2013–summer 2013	0.6244	1.2464	0.501	0.987

*P-values have been adjusted according to the single-step method using the function glht from the package multcomp in R*.

## Discussion

In this study, gastrointestinal helminths infecting captive European grey wolves (*Canis lupus lupus*) in 14 zoos distributed across Germany were identified. The most frequent parasites were hookworms, roundworms, whipworms, hairworms, taeniids, and *A. alata*, all of which have been detected in captive and/or wild wolves in Europe before (7–10, 17–21). For the interpretation of the presented data, it should be kept in mind that we were not able to match samples to single individuals. Thus, certain individuals may have contributed more than one sample per month. Accordingly, this could have lead to an over- or underestimation of true infection rates. Furthermore, it is possible that not all helminth infections were detected due to the limitations of the applied coproscopical techniques. For example, the sensitivity of the sedimentation-flotation technique for the detection of *Giardia* sp. is limited, as only *Giardia* cysts can be detected.

Hookworms showed the highest egg detection frequency (30.84% of samples) and occurred in 12 of 14 zoos. However, it has to be taken into account that strongyle eggs from feeder animals may have been subsumed under hookworm eggs, resulting in an overestimation of the actual frequency. Nevertheless, the detection rate of 30.84% in this study is similar to the percentage rate of 35.9% observed in captive wolves in Poland (10). A meta-analysis revealed an overall prevalence of 44.9% in wild wolves in Palaearctic regions (17). By contrast, Markowski (9) reported Ancylostomatidae infections in less than 2% of captive wolves in German zoos based on zoo health records. The reason for this discrepancy is unclear; however, this may be due to a lack of documentation, since hookworm infections are often subclinical and asymptomatic. In addition, anthelmintic treatment of the wolves may have played a role. Indeed, zoos in the present study which practiced anthelmintic treatment were significantly less likely to be positive for Ancylostomatidae in any given month. Nevertheless, 12 of 14 zoos were positive at least once, which requires attention since *A. caninum* possesses zoonotic potential, causing cutaneous *larva migrans* in humans (22). Thus, zoo staff should exert caution when handling substrates potentially contaminated with wolf feces, e.g., during cleaning of enclosures.

*Capillaria/Eucoleus* spp. showed the second highest egg detection rate (19.88%), and occurred in 10 of 14 zoos. Similarly, Markowski (9) found documentation of *Capillaria/Eucoleus* infection in 10.2% of wolves in German zoos. For free-ranging European gray wolves, no records of *Capillaria/Eucoleus* infections could be found in the literature. *Capillaria/Eucoleus* eggs were detected frequently in wild Ethiopian wolves (*Canis simensis*); however, it was speculated that these eggs actually represented gastrointestinal passengers, passing the intestine after consumption of infected prey (23). It cannot be excluded that this was also the case in the present study. However, it seems unlikely that the high frequency of *Capillaria/Eucoleus* eggs in the fecal wolf samples relies exclusively on intestinal transit, but the wolves were most likely infected with canine-specific species due to ingestion of embryonated eggs or earthworms containing infective larvae in their tissues. In domestic dogs, *Eucoleus aerophilus* (syn. *Capillaria aerophila*), *Capillaria putorii* (syn. *Aonchotheca putorii*), *Capillaria plica* (syn. *Pearsonema plica*), and *Capillaria hepatica* are the most common species (24). In wild foxes in Denmark, *C. plica* and *E. aerophilus* have been detected at high prevalence (25). However, since *C. plica* eggs are shed with urine, it is unlikely that these have contributed to the egg detection rate in the present study.

Roundworm prevalence in European wild wolves was reported at 17% for *T. canis* in Italy (18) and at 5.6–21.2% for *T. canis* and 1.1–13.5% for *T. leonina* in Poland (19). Here, *T. canis* was detected in 5.18% of captive wolf samples, occurring in 11 zoos, while *T. leonina* occurred in 1.25% of samples from 5 zoos. In contrast, Markowski (9) reported ascarids as the most frequently documented helminths in zoo records of captive wolves in Germany, with a prevalence of 18.3%. As transplacental and lactogenic transmission to the offspring is of particular importance in the epidemiology of *T. canis*, the lower prevalence detected in the present study may be due to the fact that the sampled population comprised only a single pup. In wild wolves, a seasonal rise in *Toxocara* egg detection frequency has been related to the presence of susceptible pups (7). In addition, changes in host immunity or the endocrinological status, e.g., related to reproduction, might lead to reactivation of dormant *Toxocara* larvae, as observed in dogs (12). Wolves show a seasonal reproductive pattern, with a mating period in winter (26). Therefore, a seasonal rise in *T. canis* egg excretion during winter was expected. However, we did not find any statistical support for this. As a zoonotic parasite, *T. canis* can cause different clinical manifestations in humans, including the *larva migrans visceralis* syndrome as well as ocular, neurological and covert toxocarosis (6). *Toxocara* eggs are not immediately infective when excreted, but may remain infective for several months after the third-stage larvae has developed, and adhere well to surfaces due to their sticky outer shell (27). Thus, contamination of enclosures may represent a source of infection for zoo staff.

Among parasites of carnivores, taeniids are also of particular zoonotic concern. Here, taeniid eggs, representing either *Taenia* spp. or *Echinococcus* spp., occurred in 8 of 14 zoos, and showed a prevalence of 3.75% among samples. Markowski (9) reported a prevalence of <2% based on zoo health records. In contrast, taeniids are the most prevalent helminths in wild wolves, with prevalences up to 64% in Spain (20), and 41.7% in Palaearctic regions (17). In contrast, Hermosilla (8) detected *Taenia* spp. with a prevalence of only 1.5% in Croatian wild wolves. The comparably low prevalence rates in zoos could be due to low prevalences in meat fed to captive wolves, low exposure of wolves to infected small mammals, or anthelmintic treatment. However, when testing the effect of diet on taeniid infections no significant influence was found, probably because all but three zoos fed game. Similarly, no significant influence of anthelmintic treatment could be detected.

Tapeworm infections are often asymptomatic in the definitive host, but may cause serious disease in the intermediate host (3). In humans, *Echinococcus granulosus* and *E. multilocularis* may cause cystic and alveolar echinococcosis, respectively, which are potentially fatal diseases (4). Less commonly, *Taenia* spp. of carnivores may also affect humans (28). In contrast to *Toxocara* eggs, taeniid eggs are immediately infective when excreted (29). Further research is needed to clarify whether captive wolves are infected with *Echinococcus* spp., to shed light on the true zoonotic relevance of the detected taeniid infections. In wild wolves in Portugal, mainly *Taenia* spp. were detected, but 1.5% of wolves also harbored *Echinococcus intermedius* (30).

*T. vulpis* and *A. alata* were both found in five zoos, and each showed a detection frequency of 3.65% among samples, which is considerably lower than detection rates reported for wild wolves. A tenfold higher *T. vulpis* detection rate (38.5%) was found in wild wolves in Poland (21), and *A. alata* rates ranged from 17.3% in Byelorussia, known today as Belarus (31), up to 80.1% in Poland (10). In recent years, *A. alata* mesocercariae have increasingly been detected in meat of wild boar in Germany (32), indicating that feeding of wild boar to wolves serves as a source of infection. As mentioned above, almost all zoos fed game to wolves. While humans may aquire *A. alata* infections after consumption of raw or undercooked meat of paratenic hosts, thus becoming a new paratenic hosts, eggs shed by definitive hosts do not constitute a source of human infection.

Apart from the abovementioned parasites, eggs of *Spirocerca lupi, A. vasorum, Opisthorchis* spp., and *Diphyllobothrium latum* were detected in the fecal samples; however, these eggs occurred at low frequencies of less than one percent. Furthermore, *Fasciola hepatica* eggs and coccidian oocysts were detected in 5.86 and 4.90% of the wolf samples, respectively. However, no statement can be made whether these originated from the wolves, from animals fed to wolves, or both as parasite host.

Apart from coccidian oocysts, no protozoan parasites were detected in this study. In contrast, *Giardia* sp. and *Cryptosporidium* sp., which have zoonotic potential, were detected in wild Croatian wolves at a prevalence of 2.1 and 1.8%, respectively (8), and in wild wolves from Canada at a prevalence of 6.8 and 1.7%, respectively (7). As mentioned above, the sensitivity of the sedimentation-flotation technique for detection of these protozoa is limited, thus, these infections may have been missed.

## Conclusion

In this study, several helminths were identified in captive wolves, which potentially compromise the wolves’ health or represent zoonotic parasites, posing a potential infection risk for zoo staff. Thus, regular anthelmintic treatment of captive wolves is recommended. Zoo staff needs to be educated in zoonotic health risks, and preventive measures to minimize these risks, such as wearing of gloves, cleaning of shoes when leaving enclosures, frequent washing of hands, and adequate disposal of feces, should be implemented or reinforced. Furthermore, carcasses and meat should be frozen before feeding to prevent wolf infections with, e.g., *A. alata* and taeniids in order to protect the health of both wolves and humans.

## Author Contributions

JB collected parts of the fecal samples and carried out laboratory work. AS and JB performed statistical analyses. CS and MB designed and coordinated the study. JB, AS, and CS drafted the manuscript. All authors participated in data interpretation and read and approved the final manuscript.

## Conflict of Interest Statement

The authors declare that the research was conducted in the absence of any commercial or financial relationships that could be construed as a potential conflict of interest. The reviewer BP and handling editor declared their shared affiliation.
